# The Efficacy of Sucralfate and Chlorhexidine as an Oral Rinse in Patients with Recurrent Aphthous Stomatitis

**DOI:** 10.1155/2014/986203

**Published:** 2014-08-14

**Authors:** Gül Soylu Özler, Şemsettin Okuyucu, Ertap Akoğlu

**Affiliations:** Department of Otorhinolaryngology, Mustafa Kemal University, 31100 Hatay, Turkey

## Abstract

*Aim*. In this study, we compared the efficacy of sucralfate suspension with chlorhexidine as an oral rinse in patients with recurrent aphthous stomatitis (RAS) in terms of pain relief and healing time. *Materials and Methods*. The subjects with a complaint of recurrent oral aphthous ulcers less than 1 cm in diameter on the first day of the occurrence of the ulcer and between 20 and 40 years were included in the study. Seventy patients completed the study. The patients were randomized into two groups as SCH group and CHX group. Changes in pain scores, healing time, and side effects of the treatments were evaluated. *Results*. The mean value of pain scores on the days after the treatment from the first day to the seventh day was significantly higher in CHX group than SCH group (*P* ≤ 0.05). On the seventh day after the treatment, the ulcers were completely reepithelialized in 23 patients in SCH group and in 19 patients in CHX group. The difference was statistically significant (*P* ≤ 0.05). In SCH group, the mean healing time of ulcers was 1.97 ± 1.56 days whereas it was 2.80 ± 3.00 days in CHX group. The difference was statistically significant (*P* ≤ 0.05). No side effects were recorded in either of the groups. *Conclusion*. Topical sucralfate suspension is an easy, safe, inexpensive, and effective treatment option for RAS to obtain pain relief and shorten the healing time of oral ulcers.

## 1. Introduction

Recurrent aphthous stomatitis (RAS) is a disease which presents as recurrent, round, shallow oral ulcerations surrounded by inflammation characterized by a break in the mucous membrane [1]. RAS is one of the most common diseases of the oral mucosa affecting 20% of the general population [2].

RAS is classified into 3 types according to the diameter of the lesion, namely, the minor, major, and herpetiform aphthous ulcerations. The most common form of RAS is minor aphthous ulcerations, and the minor form is, respectively, followed by major and herpetiform ulcerations [3]. The etiology of RAS still remains unknown. These ulcerations may be indicative of underlying systemic diseases ranging from vitamin deficiency to autoimmunity [4].

Pain is the obvious characteristic of the aphthous ulcerations causing difficulty in eating, swallowing, and speaking. To control the pain, a number of different treatment options exist including steroids, analgesics, topical anesthetics agents (lidocaine, polidocanol, benzocaine, and tetracaine), antiseptics and anti-inflammatory agents (chamomile extract solution, chlorhexidine, triclosan, and diclofenac 3% in hyaluronan), tetracycline suspension, sucralfate suspension, silver nitrate cauterization, and carbon dioxide laser [5].

Sucralfate is an agent that has been successfully used in the treatment of ulcers of the gastrointestinal tract which acts by providing a protective barrier on the surface of ulcers. Chlorhexidine is one of the most commonly prescribed agents in patients with a complaint of oral ulcers [5].

In this study we compared the efficacy of sucralfate suspension with chlorhexidine as an oral rinse in patients with RAS for pain relief and healing time.

## 2. Materials and Methods

### 2.1. Study Design

This is a randomized controlled study to compare the efficacy of sucralfate suspension with chlorhexidine as an oral rinse in the pain relief and healing time of oral aphthous ulcerations. Ethics committee approval was obtained and the study was conducted adhering to the Declaration of Helsinki. Informed consent was obtained from all subjects.

### 2.2. Study Population

The subjects attended otorhinolaryngology rooms with a complaint of recurrent oral aphthous ulcers less than 1 cm in diameter on the first day of the occurrence of the ulcer and between ages of 20 and 40 were included in the study. The patients who had an ulcer larger than 1 cm in diameter, the patients under 20 and over 50 years old, the patients who had a history of any systemic disease (ulcerative colitis, Crohn's disease, and Behçet disease), any medication (topical or systemic), and dental surgery during the previous one month were excluded.

Seventy patients completed the study. The patients were randomized into two groups. Sucralfate suspension was used 4 times a day 5 mL as an oral rinse for 1 to 2 minutes after routine mouth care and before sleep for one week in sucralfate group (SCH group) and chlorhexidine oral rinse was used 4 times a day as an oral rinse for 1 to 2 minutes after routine mouth care and before sleep for one week in chlorhexidine group (CHX group).

The clinician and patients were unaware of the treatment modality during the course of the study. The results were obtained by the same clinician. Changes in pain scores, healing time, and side effects of the treatment were evaluated. At admission the patients were asked to evaluate the severity of pain by visual analog scale (VAS). After the treatment the pain severity was evaluated on the first, third, and the seventh day. Healing time was the time needed to see the normal oral mucosa.

#### 2.2.1. Statistical Analysis

Statistical analysis was performed using the SPSS (Statistical Package for the Social Sciences) 13.0 Evaluation for Windows. Normal distribution of continues variables was tested with Kolmogorov-Smirnov test. Chi-square test was used for comparisons between categorical variables. Kruskal-Wallis test and Mann-Whitney *U* tests were used for continues variables when comparing the groups. The statistically significant level was accepted as a *P* value <0.05.

## 3. Results

Seventy patients completed the study. The mean age of SCH group and CHX group was 38.31 ± 5.40 and 38.97 ± 5.14, respectively. 51.4% of SCH group and 45.7% of CHX group were females. The groups were similar in terms of age and gender (*P* = 0.604, *P* = 0.638).

Table 1 shows the mean value of pain scores at admission and on the days after the treatment. The mean value of pain scores before the treatment was similar in both of the groups (*P* = 0.886). The mean value of pain scores on the days after the treatment from the first day to the seventh day was significantly higher in CHX group than SCH group (*P* ≤ 0.05).

On the seventh day after the treatment, the ulcers were completely reepithelialized in 23 patients (65.7%) in SCH group and in 19 patients (54.3%) in CHX group. The difference was statistically significant (*P* ≤ 0.05).

**Figure 1:**
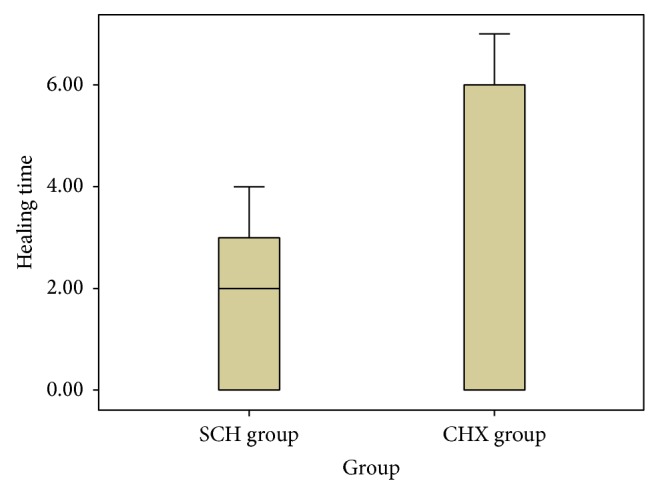
The mean healing time in ulcers in SCH group and CHX group.

In SCH group, the mean healing time of ulcers, reported by these 23 patients, was 1.97 ± 1.56 days (range 2–4). In CHX group, the mean healing time of ulcers, reported by 19 patients with healed ulcers, was 2.80 ± 3.00 days (range 4–7). The difference was statistically significant (*P* ≤ 0.05) (Figure 1).

No side effects were recorded in either of the groups.

## 4. Discussion

RAS is characterized by extremely painful aphthous ulcers causing difficulty in eating, swallowing, and speaking, so the treatment modality must obtain rapid pain relief and shorten the duration of the ulcers. Topical steroids cause pain relief and reduce ulcer frequency [6]. However, their effect on pain starts on some days and they may have some side effects [7, 8]. Topical anaesthetics reduce pain but they provide short time relief and so they must be repeated many times [9]. Chemical cauterization reduces the pain of aphthous ulcers rapidly and lasts for the duration of the ulcer [10]. Carbon dioxide laser can provide pain relief and healing immediately.

Sucralfate, an aluminium salt of sucrose octasulfate, has been successfully used in the treatment of ulcers of the gastrointestinal tract which acts by locally binding with the proteins at the base of an ulcer to provide a protective barrier [11]. Sucralfate stimulates mucus production and enhances binding of growth factors, including epidermal growth factor [12]. Sucralfate also activates both the nitric oxide and prostaglandin systems that may contribute to mucosal integrity and preservation of mucosal microcirculation [13]. Because of its antioxidant effects, sucralfate may play a role not only in the healing of damaged mucosa but also in the protection of mucosal surfaces [14].

There are previous studies reporting the positive results of the use of sucralfate suspension in patients with stomatitis, chemotherapy-induced oral mucositis, and vaginal ulceration [15–18]. In another study, Rattan et al. showed the effectiveness of sucralfate suspension in the treatment of recurrent aphthous stomatitis. They demonstrated a reduction in the healing period, duration of pain, response time to first treatment, and duration of remission in patients using sucralfate compared with placebo and antacid [19]. Alpsoy et al. showed that sucralfate therapy decreased significantly the frequency, healing time, and pain of oral ulcers and the healing time and pain of genital ulcers in patients with Behçet disease. Moreover, the effectiveness of the sucralfate on the frequency and healing time of oral ulcers continued during the posttreatment period [20].

In our study, pain scores on the days after the treatment were significantly lower in SCH group than CHX group although the mean value of pain scores before the treatment was similar. On the seventh day after the treatment, the reepithelialization of ulcers in SCH group was significantly higher than CHX group. Healing time reported in SCH group was significantly lower in CHX group. No side effects were recorded in either of the groups.

In conclusion, topical sucralfate suspension is an easy, safe, inexpensive, and effective treatment option for RAS to obtain pain relief and shorten the healing time of oral ulcers. To our knowledge, the comparison of use of sucralfate and chlorhexidine in patients with RAS has not been reported in the literature.

## Figures and Tables

**Table 1:** The mean value of pain scores at admission and on the days after the treatment.

	Before the treatment	First day after the treatment	Third day after the treatment	Seventh day after the treatment
SCH group	8.91 ± 0.78	6.42 ± 1.11	4.42 ± 0.97	0.45 ± 0.70
CHX group	8.94 ± 0.87	7.51 ± 0.74	6.14 ± 0.87	1.00 ± 1.18
*P* value	0.886	0.0001	0.0001	0.023
